# Cultural Adaptation and Validity Testing of the Portuguese Version of the Health Literacy Questionnaire (HLQ)

**DOI:** 10.3390/ijerph19116465

**Published:** 2022-05-26

**Authors:** Dulce Nascimento do Ó, Ana Rita Goes, Gerald Elsworth, João F. Raposo, Isabel Loureiro, Richard H. Osborne

**Affiliations:** 1 NOVA National School of Public Health, NOVA University Lisbon, 1169-056 Lisboa, Portugal; ana.goes@ensp.unl.pt (A.R.G.); isalou@ensp.unl.pt (I.L.); 2 NOVA National School of Public Health, Comprehensive Health Research Center, CHRC, NOVA University Lisbon, 1600-560 Lisboa, Portugal; 3 APDP-Diabetes Portugal, Rua Rodrigo da Fonseca 1, 1250-189 Lisbon, Portugal; filipe.raposo@apdp.pt; 4 Centre for Global Health and Equity, School of Health Sciences, Swinburne University of Technology, 453/469-477 Burwood Road, Hawthorn, VIC 3122, Australia; gelsworth@swin.edu.au (G.E.); rosborne@swin.edu.au (R.H.O.); 5 CEDOC-Center for the Study of Chronic Disease, NOVA Medical School, R. Câmara Pestana 6, 1150-199 Lisbon, Portugal

**Keywords:** health literacy, psychometric testing, questionnaire, diabetes, HLQ

## Abstract

Background: Health literacy is considered a determinant of self-management behaviors and health outcomes among people with diabetes. The assessment of health literacy is central to understanding the health needs of a population. This study aimed to adapt the Health Literacy Questionnaire (HLQ) to the Portuguese context and to examine the psychometric properties of a population of people with diabetes. Methods: Data were collected using a self-administrated questionnaire from 453 people with diabetes in a specialized diabetes care unit. Analysis included item difficulty level, composite scale reliability, and confirmatory factor analysis (CFA). Results: The HLQ showed that the items were easily understood by participants. Composite reliability ranged from 0.74 to 0.83. A nine-factor CFA model was fitted to the 44 items. Given the very restricted model, the fit was quite satisfactory [χ^2^_wlsmv_ = 2147.3 (df = 866), *p* = 0.001; CFI = 0.931, TLI = 0.925, RMSEA = 0.057 (90% C.I. 0.054–0.060), and WRMR = 1.528]. Conclusion: The Portuguese version of the HLQ has shown satisfactory psychometric properties across its nine separate scales in people with diabetes. Given the strong observed properties of the HLQ across cultures, languages, and diseases, the HLQ is likely to be a useful tool in a range of Portuguese settings.

## 1. Introduction

Health literacy has become an important topic across the globe in recent years, in particular for the prevention and control of non-communicable diseases (NCDs), since lifestyle-related changes, such as healthy eating, physical activity, weight control, and disease surveillance, have a central role in the reduction of premature deaths due to NCDs [1]. Diabetes is one of the four major priority NCDs targeted for action by the World Health Organization (WHO) and other authorities [1,2]. Diabetes is a serious public health problem due to its high incidence, prevalence, and serious complications, including diabetic retinopathy and peripheral vascular disease [3,4].

In 2020, the WHO stated that health literacy represents the personal knowledge and competencies that accumulate through daily activities, social interactions, and across generations. Personal knowledge and competencies are mediated by the organizational structures and availability of resources which enable people to access, understand, appraise, remember, and use information and services in ways that promote and maintain good health and well-being for themselves [5].

Diabetes control is largely dependent on individual self-management, which requires that individuals undertake complex activities including monitoring blood glucose, taking medication, eating a healthy diet, undertaking regular physical activity, and constantly adjusting treatment to the condition status. For patients to successfully undertake these complex behaviors, they must have sufficient information and skills to know what to do and make the right decisions about their self-care [4]. People in intensive insulin therapy generally require advanced diabetes-specific numeracy skills and functional health literacy to interpret food labels and calculate insulin dosage based on current blood glucose levels and carbohydrate intake. [6].

People with lower health literacy may have difficulties understanding health information and participating in healthcare consultations, which reduces their autonomy in self-care and decision-making [7,8]. Optimizing people’s health literacy has the potential to improve health and well-being and reduce health inequities [9,10]. 

Several studies among people with diabetes highlight the importance of health literacy-related knowledge [6,11,12,13,14], metabolic control [15], and better communication with health care providers [12].

In addition to the importance of health literacy for a person’s ability to manage their disease, health literacy is essential for community participation in health and planning [16]. Therefore, health literacy can be a relevant factor to consider when seeking to reduce social disparities in health outcomes [9,13,17]. 

The assessment of health literacy has been recognized as a key tool for understanding the needs and strengths within communities, organizations, or countries and for developing responses that improve access and use of health services, clinical service delivery, disease management, community participation, and policy development [16]. A variety of health literacy tools are available, but most focus on health-related reading, comprehension, and numeracy skills (known as functional health literacy), not reflecting the full range of dimensions of the health literacy concept as expressed in most contemporary definitions [16,18]. In addition, critical reviews of the properties of health literacy questionnaires show that many instruments have weak psychometric properties [19] and have difficulties in detecting differences across groups. 

The field of health literacy has recently advanced through the introduction of two multidimensional tools, the European Health Literacy Survey (HLS-EU) and the Health Literacy Questionnaire (HLQ). The HLS-EU generates three general scores developed from a review of the literature [18]. The HLQ generates nine separate scores in which the constructs were derived using a grounded validity-driven approach [19]. The HLQ was chosen for adaptation to the Portuguese context due to its utility not only in survey research but also in health literacy needs assessment and intervention development. 

The development of the HLQ included careful consideration of real-life experiences of people from the community and of health professionals. It moved beyond ideas based on early literature (focused on functional health literacy) and academic theory to represent real-world experiences of people in daily life and with a wide range of health conditions [19]. It was specifically developed to generate a diverse profile of health literacy strengths and limitations of individuals and populations. The knowledge about the needs of a population’s strengths and limitations can be used to improve clinical service delivery, community participation in health, health service planning, public health education, and policy development [16]. This comprehensive profile of health literacy allows practitioners, organizations, and governments to make decisions and plan, develop, and evaluate interventions to improve health outcomes [20].

The HLQ has been shown to have robust psychometric properties in its original version [21,22], as well as across European cultures [23,24,25,26,27,28,29,30,31] and in some Asian and African cultures [32,33,34], supporting the initial nine-factor model and thus confirming its construct validity for use in a variety of settings. To allow assessment in communities and across specific populations with the aim of adaptation of services and structures to the health needs of people struggling with disease management or prevention, the HLQ has been translated and undergone validity testing in different countries and underlies the work done in the scope of the WHO National Health Literacy Demonstration Projects, in which Portugal is involved [10]. Therefore, the adaptation and testing of the HLQ to European Portuguese is essential to demonstrate that it is culturally appropriate and psychometrically robust in this new context, providing researchers, health service providers, and policy makers with important information on its performance in this setting. In the particular case of diabetes, the HLQ is intended to be used to inform stakeholders of people’s particular health literacy needs, what supports they need, and inform the development of interventions for people dealing with their illness on a daily basis. Thus, the aim of this study was to document the psychometric properties of the Portuguese version of the HLQ in a cohort of people with diabetes. 

## 2. Materials and Methods

This study was conducted at the outpatient clinic of the Portuguese Diabetes Association (APDP). This is a national specialized diabetes care unit where most referrals are received from primary care for education and/or intensive follow-up. This institution supports people with diabetes, their families, and caregivers. It also collaborates with a range of health authorities in the development of public and social policies focused on the rights and needs of people with diabetes.

### 2.1. Setting and Participants

In this study, to determine sample size using contemporary psychometrics, we based our estimation on the simulation study conducted by Moshagen and Musch. They considered that “the range of conditions studied here, sample sizes exceeding N ≥ 300 are most likely to ensure adequate convergence of the robust WLS estimator, accurate recovery of factor loadings and covariances, and satisfactory approximation of standard errors and the (chi-square) statistic. Thus, we considered a sample > 300 ensure a robust analysis with WLSMV that provides accurate estimates of all the information from the factor analysis” [33]. Eligible participants were clinic attendees aged 18 or older with a diagnosis of diabetes. Exclusion criteria included a serious or unstable medical or psychological condition, or other condition that overtly affected cognitive ability to participate. Of 488 people approached, a total of 470 (96.3%) individuals with diabetes accepted an invitation to participate, and 453 returned completed questionnaires suitable for analysis.

Potential participants were attendees at a diabetes care unit who were approached in the waiting room. They were informed that participation was voluntary, that their information would be kept confidential, and they were provided a description of the study. Those interested were taken to a private room where they read the informed consent form and, after clarifying any concerns, provided written consent and completed the questionnaire. The researchers assisted participants who were unable to complete the questionnaire due to reading or sight difficulties or disability.

The survey was conducted between November 2018 and September 2019. Ethical and regulatory approvals were obtained from the APDP Ethics Committee (#06/2018).

### 2.2. Data Collection

In addition to the HLQ, participants were requested to provide sociodemographic (age, sex, marital status, education, living arrangements) and clinical data (self-reported health status, type of diabetes, years of living with diabetes, and metabolic control).

#### Self-Reported Health Status (SRHS)

Self-reported health status (SRHS) reflects an individual’s overall perception of their social, biological, and psychological health and has been used as an indicator of a person’s overall wellbeing in terms of social, biological and psychological health. The question has the following format: “In general, how would you rate your health?” With a five-point rating scale (Very good, Good, Fair, Poor, and Very poor).

The Health Literacy Questionnaire (HLQ) contains 44 items that cover nine separate domains (Table 1) designed to be applied in a wide range of settings (community surveys, groups, or patients) to provide a comprehensive profile of health literacy [21,22,35,36].

The first five domains are scored using a 4-point scale, from 1 = strongly disagree to 4 = strongly agree. The four remaining domains are scored on a 5-point scale for rating the level of difficulty of a task from 1 = cannot do or always difficult to 5 = always easy.

### 2.3. Translation and Cultural Adaption

The translation and cultural adaptation of the HLQ follows a strict protocol, the Translation Integrity Protocol (TIP) [37], in all languages and cultures to assure measurement quality across and within countries and to assist the development of robust evidence about the validity of decisions that can be made in new contexts and for new purposes. 

Using the TIP, the English HLQ was translated into Portuguese by two translators, guided by an extensive item intent document that provides in-depth narratives about the meaning of each element of the HLQ items, useful synonyms, and at times, what elements do not mean. The lead forward translator was a native English speaker, fluent in Portuguese, who also had extensive experience in clinical contexts. A further expert translator reviewed this initial forward translation, negotiated improvements, and a recommended forward translation was generated. This version was then further discussed by the study team (all bilingual in Portuguese and English, and with clinical or health promotion experience). This draft was then back-translated into English separately by two bilinguals. Differences in these versions were discussed and the consensus version was then reviewed by the lead author of the original English HLQ (RHO). The author, translators and research team, interrogated each item of the Portuguese translation against the original English version, the item intent and errors commonly encountered in other languages, to create the consensus version.

The final forward translation was pretested in the field using cognitive interviews with 15 people with diabetes from different age groups and across education levels. Each respondent was carefully observed while filling out the questionnaire, and the interviewer reviewed the items with the respondents and asked specific questions to clarify reactions and response options. Prompt questions included: “What were you thinking about when you answered this question?” and “Why did you chose that response?”.

In addition, participants were asked to read the questions out loud and explain the meaning of what they had just read, while the interviewer recorded any doubts or difficulties. This process assisted in understanding the need for changes or improvements to the items. After detailed analysis of these data by the research team, the final translated questionnaire was confirmed. 

### 2.4. Data Analysis

Sociodemographic and clinical characteristics of participants were summarized by median (interquartile range) or percentages. Missing values were determined by descriptive statistics performed for each item. If there were more than 2 missing values for scales with 4–5 questions and fewer than 3 missing values from the scale with 6 items, the responses were not included in the results for that participant on that scale. 

Item difficulty was determined by descriptive statistics consistent with previous HLQ studies [24,26,33]. For the first five domains, the difficulty level was calculated as the number of strongly disagree/disagree responses divided by the number of agree/strongly agree responses. For the remaining four domains, difficulty was calculated as the number of cannot do or always difficult/usually difficult/sometimes difficult responses divided by the number of usually easy/always easy responses. 

Reliability was examined using Cronbach’s α and composite reliability. Given that the HLQ scales were specified a priori, factor structure was tested using confirmatory factor analysis (CFA) to determine if the original hypothesized nine constructs were evident in the data. A nine factor CFA model was fitted to the 44 items and one-factor CFA models were fitted to the data for each previously confirmed scale [21]. Mplus was used to calculate the weighted least squares mean and variance adjusted (WLSMV) estimator, the unstandardized and standardized factor loadings, an estimate of the variance in the measured variable explained by the latent variable (R^2^), and the associated standard errors, together with fit statistics (χ^2^, CFI—comparative fit index; TLI—Tucker–Lewis index; RMSEA—root mean square error of approximation; WRMR—weighted root mean square residual). In our study we used the indicative threshold values for the tests of “close fit” (CFI > 0.95; TLI > 0.95; WRMR < 1000; RMSEA < 0.06 and values < 0.08 suggests a reasonable model–data fit [38,39]). Inter-factor correlations > 80 are indicators of a potential lack of discriminant validity in the second part of HLQ [40].

Mean differences of HLQ scores across a range of sociodemographic and clinical variables were determined using one-way analysis of variance (ANOVA). The effect size eta squared (η^2^) was provided by SPSS and was calculated using the sum of squares of the effect divided by the total sum of squares (η^2^ = SS between groups/SS total) [40,41,42]. Cohen (1988) has provided benchmarks to define small (η^2^ = 0.01), medium (η^2^ = 0.06), and large (η^2^ = 0.14) effects. Their 95% confidence intervals (CI) were determined based on a non-central F distribution according to Karl Wuensch’s work [43]. 

Analyses were conducted using IBM SPSS Statistics version 23 (IBM, New York, NY, USA) and Mplus version 7 (Muthén & Muthén, Los Angeles, CA, USA).

## 3. Results

### 3.1. Cognitive Testing

During the first set of eight interviews, almost all items were well understood. Only minor reformulation needs were identified. The Portuguese expression for “health information” was found to be hard to understand and participants suggested the Portuguese expression for “information about health”. In Portuguese culture, the word “discuss” may have a conflicted meaning, so it was suggested to change it to “talk”. Regarding the design of the questionnaire, some participants found it difficult to read the response options positioned vertically, suggesting a change to a horizontal presentation. After these minor changes, we conducted a second set of seven interviews and no further improvements were identified.

### 3.2. Missing Values

Across the 453 respondents, there were few missing responses (0.4 to 0.9%), indicating the high acceptability of the items to respondents. The highest missing number was found in the items “I set my own goals about health and fitness” and “I have at least one healthcare provider who knows me well” which had 3 missing responses. The items “When I feel ill, the people around me really understand what I am going through” and “I spend quite a lot of time actively managing my health” both had 2 missing responses.

### 3.3. Sociodemographic Characteristics of Participants

Table 2 shows the sociodemographic and health characteristics of the participants. The median age was 61 years. More than half were men (51.2%), 83.2% lived in cohabitation, 47% were retired, 40% employed, and 51.7% had nine or fewer years of school. Regarding self-reported health status, 73.7% considered their health to be fair or poor and 74.6% of the participants reported having type 2 diabetes (T2D). Only 23.8% reported having been living with diabetes for 10 or fewer years. The median glycated haemoglobin (HbA1c) was 7.9% (extremes 5.1–14.2).

### 3.4. Psychometrics Properties

The psychometric properties of the individual HLQ scales are described in Table 3. The estimates of composite reliability were satisfactory, ranging from 0.74 to 0.83. The lowest reliability estimate was for 4. *Social support for health* and the highest for scale 6. *Ability to actively engage with health care providers*. Cronbach’s α provided similar results (Table 3).

Considering the one-factor models, the model fit for all scales was generally good except for 5. *Appraisal of health information*. For this scale, RMSEA was high (>0.08), but CFI and TLI were above 0.95 and WRMR < 1.000 (Table 3). For each scale, factor loadings were satisfactory, with 37 of the 44 items showing factor loadings above 0.60 (range 0.60–0.80).

A nine factor CFA model was fitted to the 44 items. Given the very restricted nature of the model, the fit was quite satisfactory: χ^2^_wlsmv_ = 2147.353 (df = 866), *p* = 0.001; CFI = 0.931, TLI = 0.925, RMSEA = 0.057 (90% C.I. 0.054–0.060), and WRMR = 1.528. While the CFI and TLI are lower than the pre-specified cut-off and the WRMR is higher, this is not surprising given the large number of parameters in the model set precisely to 0.0. [19]. The ranges of factor loadings in this model were: 1. *Feeling understood and supported by healthcare providers* (0.74–0.88); 2. *Having sufficient information to manage my health* (0.76–0.86); 3. *Actively managing my health* (0.65–0.88); 4. *Social support for health* (0.40–0.89); 5. *Appraisal of health information* (0.65–0.79); 6. *Ability to actively engage with healthcare providers* (0.67–0.86); 7. *Navigating the healthcare system* (0.65–0.77); 8. *Ability to find good health information* (0.72–0.77) and 9. *Understanding health information well enough to know what to do* (0.42–0.78).

The inter-factor correlations presented in Table 4 show the discrimination between the scales in part 1 and part 2. The inter-factor correlations in the nine-factor model range from 0.283 (scales 3 and 8) to 0.891 (6 and 7). The results of the correlations demonstrated the discrimination between the scales in part 1 (ranging from 0.283 to 0.762). However, in part 2, the discrimination between the scales was less evident (range 0.718–0.891), with several inter-factor correlations above 0.80.

### 3.5. Item Difficulty

The difficulty level of the translated HLQ items is presented in Table 5. For scales 1–5 (part 1 of the questionnaire), scale 1. *Feeling understood and supported by healthcare providers* showed the lowest difficulty level, with an average item difficulty of 0.08. That is to say, on average across the five items, 8% of respondents indicated that they strongly disagreed or disagreed with the items in this scale. Scale 5. *Appraisal of health information* showed the highest difficulty level (0.29). For scales 6–9, scale 6. *Ability to actively engage with healthcare providers* had the lowest difficulty level (0.24) and scale 8. *Ability to find good health information* (0.42) and 7. *Navigating the healthcare system* (0.40) had the highest difficulty levels.

### 3.6. Scales Scores

The descriptive data for the nine scales of HLQ are represented in Table 6. For the first five dimensions, the highest score was found for scale 1. *Feeling understood and supported by healthcare providers* and the lowest for scale 5. *Appraisal of health information* and 3. *Actively managing my health*. For part 2, the highest score was found for scale 6. *Ability to actively engage with healthcare providers* and the lowest for scale 8. *Ability to find good health information*.

### 3.7. Health Literacy Dimensions, Social and Clinical Variables, and Health Status

Table 7 shows the HLQ scale scores according to sociodemographic and health variables. Men and women have very similar scores. Significantly higher scores were observed for people aged 18 to 44 in all domains except domain 6. *Ability to actively engage with healthcare providers*. Participants with university-level qualifications had significantly higher health literacy levels across most domains of the HLQ than participants with the lowest education. Employed respondents had higher scores compared with unemployed in domains 4, 6, 8 and 9. Those who lived with someone had significantly higher scores in domains 4 and 8. Participants with type 1 diabetes had significantly higher scores in all domains but one, domain 6. *Ability to actively engage with healthcare providers*. We point out that, in this sample, 46.2% of people with T1D have a university-level education. Patients with better metabolic control (HbA1c < 6.5%) had higher scores in domains 1, 3, and 4 compared with people with the worst metabolic control (HbA1c > 8.5%). Regarding self-reported health, respondents rating themselves as having good (results of excellent, very good or good status) and fair health status had significantly higher scores in all domains but domain 1 compared with those with poor health status.

## 4. Discussion

The Portuguese version of the HLQ has strong psychometric properties for use among people with diabetes in clinical practice and for the advancement of health literacy research. These data provide important evidence about the potential of the HLQ to provide useful information to policy makers, researchers, and healthcare providers to support efforts to improve health and equity among people with diabetes in Portugal. 

This version was produced through rigorous linguistic, cultural, and measurement adaptation methods. The items were found to be well understood by participants. Using a highly restrictive confirmatory factor analysis model, the original 9-factor model was confirmed (with no cross loadings or correlated residuals), all the scales had acceptable reliability, and expected differences between demographic subgroups were observed. 

The data also gives us important evidence that the HLQ may have specific utility for the management of diabetes. The health literacy dimension related to the skill of actively managing health (scale 3) was clearly correlated with HbA1c status and the time people had been living with diabetes (people recently diagnosed had lower active management). Lower health literacy was strongly associated with self-reported health status, particularly for people with low scores on understanding health information (scale 9). Across all scales, except engagement with healthcare providers (scale 6), people with Type 2 diabetes reported lower health literacy. Clear patterns of lower health literacy on most scales were observed for older age groups and those with lower education. 

While no causal conclusions can be made, due to the cross-sectional nature of the study, these data indicate that the HLQ has the potential to identify particular subgroups at risk of lower health literacy, who have particular challenges with the management of their condition, and who may benefit from specific health literacy-informed services and supports.

This instrument has been specifically designed to evaluate the needs of individuals, communities, and services, and applied at a national level. The data generated are expected to assist with identifying ways to improve individual and community health literacy. Also, it can be used to measure outcomes of public health and clinical interventions designed to improve health literacy [21]. Importantly, in field tests, Portuguese interviewees clearly understood most of the items according to the specific item intents and after revisions, no further difficulties were found. Most participants considered the final version questionnaire easy and quick to complete, taking 10–15 min for most people, including older people, to complete. However, some participants did find it challenging to decide what sources of information should be considered in 2 items that referred to “information from different sources”. In scale 7. *Navigating the health care system*, item 7.4 *Make sure you find the right place to get the health care you need* had a high difficulty score. Given that in Portuguese public health care services, people are not usually given the possibility of choosing their health care providers. The primary care physician is the gatekeeper and makes decisions regarding referral to other health care services. The scales with the highest difficulty scores were 5, 7, and 8 and the lowest were 1 and 6. The difficulty level we obtained is somewhat different to other language adaptations. In a Danish study, the scales 2 and 4 were the easiest and the scales 5 and 7 the hardest [24]. In the Ghanaian language the scales 3 and 8 had lowest difficulty and scales 1 and 7 the highest difficulty [33]. In the French study, the scales 1 and 9 were easiest and 2 and 7 the hardest [26]. It is likely that the complexity, resources, and culture of each country influence the way health care and health information are perceived [30,31]. Regarding the difficulty scores, within each scale, the Portuguese items generally have a range of difficulty that should enable the Portuguese HLQ to capture differences between individuals.

The data obtained with the Portuguese version of the HLQ suggests satisfactory construct validity for our population. The original dimensional structure with nine scales was confirmed, attesting to the conceptual robustness of the HLQ [21]. The majority of the Portuguese HLQ items loaded highly on their respective factors, with only 3 items loading below 0.6. One problematic item was 9.2, *Accurately follow instructions from healthcare providers* with a low loading (0.26). Compared with the other items in the scale, this one has a stronger focus on following instructions, whereas the 3 other items are about reading, writing, and filling in forms. The other item in the scale, with a lower loading of 0.57, also has an element of understanding healthcare providers. The process of understanding is often very complex for people with diabetes, given the many treatments they may require, and nonadherence is frequently observed. Item 9.2 may be read as having a strong connection with the complex self-management tasks and decision making and not only with the ability to understand health information. Indeed, in diabetes care, the treatment involves not only medication, but also diet, exercise, self-monitoring of blood glucose, preventive foot care, periodic eye examinations, and avoiding high risk behaviors (e.g., smoking, alcohol) with frequent adjustments to the regimen.

The Portuguese version of the HLQ demonstrated satisfactory reliability ranging from 0.74 to 0.83, which is consistent with the scores found in previous studies with the HLQ, namely the Brazilian (0.67 to 0.82) [23], Danish (0.77–0.87) [24], Slovak (0.73 to 0.84) [25], French (0.77 to 0.91) [26], German (0.77 to 0.91) [27], Norwegian (0.71 to 0.87) [28], Dutch (0.83 to 0.94) [29], Urdu (0.84 to 0.91) [32], Ghanaian (0.66 to 0.82) [33] Nepalese studies (0.74 to 0.88) [34].

Inter-factor correlations suggested good discriminant validity between Part 1 scales, but there are some high intercorrelations in Part 2. While they qualitatively represent distinct concepts, the high correlations (up to 0.89) suggest that there may be some higher-order concepts linking the scales (e.g., self-efficacy, general capacity to interact positively and effectively with the health care system). Strong associations between these scales have been found in previous studies with the HLQ [21,24,25,26,27,29,31] and deserve further exploration in future studies. In addition, in the French HLQ study, the cultural variation across European cultures was analyzed as a potential confounder of the item and scale difficulty data [26]. These authors recommend, given the risk of bias, delaying the comparison of health literacy scores between cultures, prior to the generation of robust evidence.

The HLQ scores were associated with sociodemographic and clinical characteristics in expected ways. Education was clearly associated with health literacy levels, except for the scales most strongly linked to social issues (i.e., 4. *Social support for health* and 6. *Ability to actively engage with healthcare providers*). Younger participants reported higher scores in all dimensions, except for 6. *Ability to actively engage with healthcare providers*. Compared with people who lived alone, those who cohabitated had higher scores in 4. *Social support for health* and 8. *Ability to find good health information*, underlying the importance of significant others for health literacy. Overall, older age, low education level, living alone, and being unemployed were associated with lower health literacy scores. This finding is consistent with prior Portuguese studies and the Health Literacy Population Survey Project 2019–2021 where the results suggest higher health literacy is associated with younger age, being male, with greater economic capacity, education, and being employed. [44,45].

Regarding clinical characteristics, participants with better diabetes control (HbA1c < 6.5%) showed higher scores in domain 1. *Feeling understood and supported by healthcare providers*, 3. *Actively managing my health*, and 4. *Social support for health*. These results are similar to a prior study using the HLQ with people with type 1 diabetes [46]. Notwithstanding that this is correlational data, and causal directions cannot be determined, this link to harder biological parameters of people with diabetes is potentially useful for identifying potential targets for interventions. These aspects are considered essential for self-management, participation in medical decision-making and diabetes control [5,7,13]. Other studies showed that people with lower levels of health literacy were more likely to report worse communication with health professionals [21] and less willingness to participate in the clinical decision-making process [15].

Lastly, individuals with better self-rated health had significantly higher scores in all domains, except domain 1. This finding is similar to other studies that highlight health literacy as a determinant for good health perception [24,47]. It is also in accordance with theoretical models that propose health literacy to be an antecedent of health outcomes [48]. A strength of this study is that the validation sample was drawn from an outpatient clinic for people with diabetes that accepts people with diabetes from all over the country. The recruitment method ensured a high level of participation from people with diverse background characteristics. The sample recruited has somewhat higher education, age, and employment status than the general Portuguese population. However, it is likely to be a reasonable representation of people with type 2 diabetes. A potential limitation is that the Portuguese Diabetes Association, with strong investments in therapeutic education, may have biased the sample towards people with more exposure of self-management support compared with the general population of people with diabetes. Future research should include different settings, such as primary care, hospitals, and different community settings.

## 5. Conclusions

The present study demonstrates that the Portuguese version of the HLQ has acceptable construct validity and reliability for use among people with various types of diabetes. As such, the HLQ is likely to be useful to advance health literacy research and practice in this setting. Given that diabetes is a typical chronic condition, the HLQ is likely to be useful in other diseases and among the general population. However, further validity testing is warranted. The HLQ is now being applied in the Ophelia (Optimising Health Literacy and Access) process to co-design diabetes interventions with stakeholders. The current study provides critical validity testing data to inform stakeholders regarding the use of the HLQ to assist health care providers, planners, and policymakers in adjusting the planning, design, and evaluation of interventions to promote health and health literacy.

## Figures and Tables

**Table 1:** Health Literacy Questionnaire (HLQ) scales with high and low descriptors of each construct [21].

Low Level of the Construct	High Level of the Construct
**1. Feeling understood and supported by healthcare providers**	
People who are low on this domain are unable to engage with doctors and other healthcare providers. They don’t have a regular healthcare provider and/or have difficulty trusting healthcare providers as a source of information and/or advice.	Has an established relationship with at least one healthcare provider who knows them well and who they trust to provide useful advice and information and to assist them to understand information and make decisions about their health.
**2. Having sufficient information to manage my health**	
Feels that there are many gaps in their knowledge and that they don’t have the information they need to live with and manage their health concerns.	Feels confident that they have all the information that they need to live with and manage their condition and to make decisions.
**3. Actively managing my health**	
People with low levels don’t see their health as their responsibility, they are not engaged in their healthcare and regard healthcare as something that is done to them.	Recognise the importance and are able to take responsibility for their own health. They proactively engage in their own care and make their own decisions about their health. They make health a priority.
**4. Social support for health**	
Completely alone and unsupported for health.	A person’s social system provides them with all the support they want or need for health.
**5. Appraisal of health information**	
No matter how hard they try, they cannot understand most health information and get confused when there is conflicting information.	Able to identify good information and reliable sources of information. They can resolve conflicting information by themselves or with help from others.
**6. Ability to actively engage with healthcare providers**	
Are passive in their approach to healthcare, inactive i.e., they do not proactively seek or clarify information and advice and/or service options. They accept information without question. Unable to ask questions to get information or to clarify what they do not understand. They accept what is offered without seeking to ensure that it meets their needs. Feel unable to share concerns. The do not have a sense of agency in interactions with providers.	Is proactive about their health and feels in control in relationships with healthcare providers. Is able to seek advice from additional healthcare providers when necessary. They keep going until they get what they want. Empowered.
**7. Navigating the healthcare system**	
Unable to advocate on their own behalf and unable to find someone who can help them use the healthcare system to address their health needs. Do not look beyond obvious resources and have a limited understanding of what is available and what they are entitled to.	Able to find out about services and supports so they get all their needs met. Able to advocate on their own behalf at the system and service level.
**8. Ability to find good health information**	
Cannot access health information when required. Is dependent on others to offer information.	Is an ‘information explorer’. Actively uses a diverse range of sources to find information and is up to date.
**9. Understanding health information well enough to know what to do**	
Has problems understanding any written health information or instructions about treatments or medications. Unable to read or write well enough to complete medical forms.	Is able to understand all written information (including numerical information) in relation to their health and able to write appropriately on forms where required.

**Table 2:** Sociodemographic and clinical characteristics of participants.

Characteristic	Percentage *	*n*
Age, years median (IQR)	61 (18)	453
Age, years (max-min)	22–96	
Sex (%)		453
Female	48.8	221
Cohabitation status (%)		450
Live alone	15.9	72
Employment status (%)		452
Employed	40.6	184
Unemployed	47.0	213
Other	12.1	45
Education level (%)		451
<4 years	29.8	135
4–9 years	21.9	99
10–12 years	23.8	108
University	24.1	109
Self-reported health status		
Excellent	1.3	6
Very good	13.1	14
Good	21.4	97
Fair	53.6	243
Poor	20.1	91
Type of diabetes		
Type 1 Diabetes	23.0	104
Type 2 Diabetes	74.6	338
Other type	2.4	11
Years living with diabetes		453
<10 years	23.8	108
10–19 years	37.1	168
>20 years	39.1	177
Metabolic control HbA1c medium (IQR)	7.9 (2.0)	441
Metabolic control HbA1c (max-min)	5.1–14.2	441

* Percentage of respondents unless otherwise stated. IQR: interquartile range.

**Table 3:** Psychometric properties of the Portuguese translation of the Health Literacy Questionnaire among people with diabetes in Portugal.

	Factor Loading (95% CI)	R^2^	Cronbach Alpha	Composite Reliability (95% CI)
1. Feeling understood and supported by healthcare providers			0.81	0.79 (0.77–0.84)
I have at least one healthcare provider who…	0.79 (0.74–0.84)	0.62		0.74 (0.72–0.80)
I have at least one healthcare provider I can…	0.79 (0.75–0.84)	0.63		0.73 (0.71–0.79)
I have the healthcare providers I need…	0.77 (0.73–0.81)	0.59		0.75 (0.74–0.81)
I can rely on at least one healthcare provider	0.86 (0.82–0.91)	0.75		0.70 (0.68–0.77)
Model fit − χ^2^_wlsmv_ (2) = 5.859, *p* = 0.0534, CFI = 0.998, TLI = 0.995, RMSEA = 0.065, WRMR = 0.359				
2. Having sufficient information to manage my health			0.80	0.80 (0.77–0.83)
I feel I have good information…	0.69 (0.64–0.75)	0.48		0.79 (0.76–0.82)
I have enough information to help me deal…	0.86 (0.81–0.90)	0.73		0.74 (0.70–0.78)
I am sure I have all the information I…	0.80 (0.76–0.85)	0.64		0.74 (0.70–0.78)
I have all the information I…	0.82 (0.77–0.86)	0.66		0.75 (0.71–0.79)
Model fit − χ^2^_wlsmv_ (2) = 7.853, *p* = 0.0197, CFI = 0.997, TLI = 0.992, RMSEA = 0.080, WRMR = 0.461				
3. Actively managing my health			0.79	0.79 (0.76–0.82)
I spend quite a lot of time actively…	0.71 (0.65–0.77)	0.51		0.76 (0.73–0.80)
I make plans for what I…	0.70 (0.64–0.75)	0.48		0.75 (0.72–0.79)
Despite other things in my life, I…	0.80 (0.76–0.85)	0.65		0.75 (0.71–0.78)
I set my own goals about health…	0.68 (0.61–0.74)	0.46		0.76 (0.72–0.80)
There are things that I do regularly…	0.74 (0.69–0.80)	0.55		0.76 (0.72–0.79)
Fit with 1 correlated residual − χ^2^_wlsmv_ (4) = 4.152, *p* = 0.0197, CFI = 1.000, TLI = 1.000, RMSEA = 0.009, WRMR = 0.257				
4. Social support for health			0.74	0.74 (0.71–0.78)
I can get access to several people who…	0.70 (0.64–0.77)	0.49		0.70 (0.65–0.74)
When I feel ill, the people around me…	0.57 (0.50–0.65)	0.33		0.73 (0.69–0.77
If I need help, I have plenty of people…	0.83 (0.78–0.88)	0.69		0.66 (0.61–0.71)
I have at least one person who can come-…	0.51 (0.43–0.59)	0.26		0.73 (0.69–0.77)
I have strong support from family…	0.70 (0.64–0.76)	0.49		0.68 (0.64–0.73)
Fit with 1 correlated residual- χ^2^_wlsmv_ (4) = 10.007, *p* = 0.0403, CFI = 0.995, TLI = 0.987, RMSEA = 0.058, WRMR = 0.453				
5. Appraisal of health information			0.76	0.77 (0.73–0.80)
I compare health information from…	0.78 (0.73–0.83)	0.60		0.70 (0.66–0.75)
When I see new information about health, I…	0.73 (0.70–0.79)	0.53		0.72 (0.67–0.76)
I always compare health information from…	0.84 (0.79–0.89)	0.70		0.69 (0.64–0.73)
I know how to find out if the health information…	0.55 (0.47–0.62)	0.30		0.75 (0.72–0.79)
I ask healthcare providers about the quality…	0.53 (0.46–0.61)	0.28		0.76 (0.72–0.79)
Fit with 1 correlated residual- χ^2^_wlsmv_ (4) = 23.483, *p* = 0.0001, CFI = 0.987, TLI = 0.968, RMSEA = 0.104, WRMR = 0.623				
*PART2-Scale 6–9:*				
6. Ability to actively engage with healthcare providers			0.83	0.83 (0.80–0.85)
Make sure that healthcare providers understand…	0.70 (0.64–0.76)	0.49		0.81 (0.79–0.84)
Feel able to discuss your health concerns with…	0.74 (0.68–0.79)	0.55		0.81 (0.78–0.83)
Have good discussions about your health…	0.80 (0.75–0.85)	0.64		0.79 (0.76–0.82)
Discuss things with healthcare providers…	0.76 (0.70–0.81)	0.57		0.79 (0.76–0.82)
Ask healthcare providers questions to get…	0.80 (0.75–0.86)	0.65		0.78 (0.75–0.81)
Fit with 1 correlated residual − χ^2^_wlsmv_ (4) = 6.002, *p* = 0.1990, CFI = 0.999, TLI = 0.998, RMSEA = 0.033, WRMR = 0.294				
7. Navigating the healthcare system			0.80	0.81 (0.78–0.83)
Find the right health care	0.57 (0.49–0.64)	0.32		0.80 (0.77–0.83)
Get to see the healthcare providers you…	0.73 (0.68–0.76)	0.53		0.77 (0.74–0.80)
Decide which healthcare provider you need…	0.74 (0.69–0.79)	0.55		0.77 (0.73–0.80)
Make sure you find the right place to get the…	0.82 (0.79–0.86)	0.68		0.75 (0.71–0.79)
Find out which healthcare services you are…	0.69 (0.64–0.75)	0.48		0.78 (0.74–0.81)
Work out what the best care is…	0.60 (0.54–0.66)	0.36		0.79 (0.76–0.82)
Model fit − χ^2^_wlsmv_ (9) = 4.736, *p* = 0.8567, CFI = 1.000, TLI = 1.003, RMSEA = 0.000, WRMR = 0.235				
8. Ability to find good health information			0.82	0.82 (0.79–0.84)
Find information about health…	0.75 (0.70–0.80)	0.56		0.79 (0.75–0.82)
Find health information from several…	0.77 (0.72–0.82)	0.59		0.77 (0.74–0.81)
Get information about health so you are up…	0.72 (0.65–0.78)	0.51		0.78 (0.75–0.82)
Get health information in words you understand…	0.70 (0.64–0.75)	0.49		0.79 (0.76–0.82)
Get health information by yourself	0.76 (0.71–0.81)	0.58		0.78 (0.75–0.81)
Model fit − χ^2^_wlsmv_ (5) = 12.8289, *p* = 0.0250, CFI = 0.996, TLI = 0.992, RMSEA = 0.059, WRMR = 0.422				
9. Understanding health information well enough to know what to do			0.75	0.79 (0.76–0.82)
Confidently fill medical forms in the…	0.70 (0,64–0,76)	0.49		0.75 (0.71–0.79)
Accurately follow instructions from…	0.26 (0.18–0.35)	0.07		0.81 (0.78–0.84)
Read and understand written health…	0.88 (0.84–0.90)	0.78		0.67 (0.62–0.72)
Read and understand all the information…	0.82 (0.76–0.84)	0.67		0.70 (0.66–0.75)
Understand what healthcare providers…	0.57 (0.51–0.64)	0.33		0.78 (0.74–0.81)
Fit with 1 correlated residual- χ^2^_wlsmv_ (4) = 5.027. *p* = 0.2845. CFI = 0.999. TLI = 0.999, RMSEA = 0.024, WRMR = 0.279				

**Table 4:** Inter-factor correlations among the nine Health Literacy Questionnaire scales derived from a nine-factor confirmatory factor analysis model.

	Part 1					Part 2		
Scale	1	2	3	4	5	6	7	8
2. Having sufficient information to manage my health	0.762							
3. Actively managing my health	0.439	0.549						
4. Social support for health	0.752	0.674	0.412					
5. Appraisal of health information	0.548	0.659	0.646	0.479				
6. Ability to actively engage with healthcare providers	0.654	0.554	0.347	0.540	0.390			
7. Navigating the healthcare system	0.616	0.620	0.367	0.540	0.450	0.891		
8. Ability to find good health information	0.465	0.614	0.283	0.412	0.581	0.718	0.888	
9. Understanding health information well enough to know what to do	0.419	0552	0.321	0.398	0.423	0.762	0.814	0.889

**Table 5:** Difficulty level of the translated Health Literacy Questionnaire (HLQ) in a Portuguese population *.

	Obs (*n* = 453)	Missing *n* (%)	Median	Mean (SD)	Difficult Level % (95% CI) ^a^
***Part 1: How strongly you disagree or agree with the following statements (Strong disagree, disagree, agree, strongly agree)***					
**1-Feeling understood and supported by healthcare providers**					
I have at least one healthcare provider who…	453	0	3	3.21 (0.62)	9.7 (7.1–12.8)
I have at least one healthcare provider I can…	453	0	3	3.22 (0.54)	7.3 (5.1–10.1)
I have the healthcare providers I need…	453	0	3	3.19 (0.52)	7.1 (4.9–9.8)
I can rely on at least one…	453	0	3	3.20 (0.58)	6.8 (4.7–9.6)
**2-Having sufficient information to manage my health**					
I feel I have good information…	450	3 (0.7)	3	3.05 (0.58)	11.8 (8.9–15.1)
I have enough information to help me deal…	453	0	3	3.09 (0.60)	15.7 (12.4–19.4)
I am sure I have all the information I…	453	0	3	2.88 (0.62)	24.3 (20.4–28.4)
I have all the information I…	453	0	3	3.04 (0.60)	20.5 (16.9–24.5)
**3-Actively managing my health**					
I spend quite a lot of time actively…	451	2 (0.4)	3	2.75 (0.72)	33.7 (29.3–38.3)
I make plans for what I…	453	0	3	2.85 (0.59)	24.9 (21.0–29.2)
Despite other things in my life...	453	0	3	2.80 (0.59)	26.7 (22.7–31.0)
I set my own goals about health…	449	4 (0.9)	3	2.87 (0.68)	28.7 (24.6–33.2)
There are things that I do regularly…	453	0	3	2.92 (0.60)	21.2 (17.5–25.2)
**4-Social support for health**					
I can get access to several people who…	453	0	3	3.15 (0.62)	11.0 (8.3–14.3)
When I feel ill, the people around me…	451	2 (0.4)	3	2.94 (0.68)	21.1 (17.4–25.1)
If I need help, I have plenty of people…	453	0	3	3.03 (0.72)	18.3 (14.9–22.2)
I have at least one person who can come…	453	0	3	3.06 (0.78)	17.7 (14.3–21.5)
I have strong support from family…	453	0	3	3.20 (0.73)	11.9 (9.1–15.3)
**5-Appraisal of health information**					
I compare health information from…	453	0	3	2.74 (0.68)	32.7 (28.4–37.2)
When I see new information about health. I…	453	0	3	2.91 (0.66)	22.7 (19.0–26.9)
I always compare health information from…	453	0	3	2.72 (0.65)	34.9 (30.5–39.5)
I know how to find out if the health…	453	0	3	2.81 (0.65)	28.9 (24.8–33.3)
I ask healthcare providers about the quality…	453	0	3	2.87 (0.68)	26.5 (22.5–30.8)
	**Obs** **(*n* = 453)**	**Missing** ***n* (%)**	**Median**	**Mean (SD)**	**Difficult Level %** **(95% CI) ^b^**
***Part 2: How easy or difficult the following tasks are for you to do now (cannot do, very difficult, quite difficult, quite easy, very easy)***					
**6-Ability to actively engage with healthcare providers**					
Make sure that healthcare providers understand…	453	0	4	3.81 (0.77)	27.2 (23.2–31.5)
Feel able to discuss your health concerns with…	453	0	4	4.03 (0.75)	16.8 (13.5–20.5)
Have good discussions about your health…	453	0	4	3.85 (0.80)	23.8 (20.0–28.0)
Discuss things with healthcare providers…	453	0	4	3.79 (0.75)	28.3 (24.2–32.6)
Ask healthcare providers questions to get…	453	0	4	3.80 (0.78)	24.9 (21.0–29.2)
**7-Navigating the healthcare system**					
Find the right health care	453	0	4	3.49 (0.81)	47.5 (42.8–52.2)
Get to see the healthcare providers you…	453	0	4	3.64 (0.86)	38.9 (34.4–43.4)
Decide which healthcare provider you need…	453	0	4	3.70 (0.81)	32.9 (28.7–37.4)
Make sure you find the right place to get the…	453	0	4	3.64 (0.82)	37.7 (33.4–42.3)
Find out which healthcare services you are…	453	0	4	3.44 (0.85)	50.1 (45.4–54.8)
Work out what the best care is…	453	0	4	3.664 (0.79)	35.3 (30.9–39.9)
**8-Ability to find good health information**					
Find information about health…	453	0	4	3.65 (0.77)	35.8 (31.3–40.4)
Find health information from several…	453	0	4	3.43 (0.91)	49.2 (44.5–53.9)
Get information about health so you are up…	453	0	4	3.63 (0.80)	36.4 (32.0–41.0)
Get health information in words you understand…	453	0	4	3.53 (0.89)	44.4 (30.7–49.1)
Get health information by yourself	453	0	4	3.40 (0.98)	46.1 (41.5–50.9)
**9-Understanding health information well enough to know what to do**					
Confidently fill medical forms in the…	453	0	4	3.74 (0.98)	28.3 (24.2–32.6)
Accurately follow instructions from…	453	0	4	3.68 (0.74)	38.4 (33.9–43.1)
Read and understand written health…	453	0	4	3.56 (0.95)	37.3 (32.8–41.9)
Read and understand all the information…	453	0	4	3.45 (1.02)	44.8 (40.2–49.5)
Understand what healthcare providers…	453	0	4	4.02 (0.66)	14.1 (11.1–17.7)

^a^ Difficulty level was calculated as the number of respondents choosing disagree and strongly disagree divided by the number choosing agree or strongly agree. ^b^ Difficulty level was calculated as the number of respondents choosing cannot do, very difficult or quite difficult dived by the number choosing quite easy and very easy. * Items are truncated. Complete items are available from the copyright holder HLQ-info@swin.edu.au.

**Table 6:** Health Literacy Questionnaire (HLQ) scales scores.

	Mean Score (SD)	Min Score	Max Score	(95% CI)
Part 1-Range 1 (lowest) to 4 (highest)				
1. Feeling understood and supported by healthcare providers	3.21 (0.47)	1.75	4.00	3.16–3.25
2. Having sufficient information to manage my health	2.98 (0.48)	1.50	4.00	2.94–3.03
3. Actively managing my health	2.83 (0.46)	1.20	4.00	2.78–2.87
4. Social support for health	3.08 (0.50)	1.20	4.00	3.03–3.12
5. Appraisal of health information	2.81 (0.47)	1.00	4.00	2.77–2.86
Part 2-Range 1 (lowest) to 5 (highest)				
6. Ability to actively engage with healthcare providers	3.86 (0.59)	2.00	5.00	3.80–3.91
7. Navigating the healthcare system	3.60 (0.58)	1.83	5.00	3.55–3.60
8. Ability to find good health information	3.53 (0.66)	1.20	5.00	3.46–3.59
9. Understand health information well enough to know what to do	3.69 (0.62)	1.40	5.00	3.63–3.75

SD-Standard deviation; CI-confidence interval.

**Table 7:** Relationship between Health Literacy Questionnaire (HLQ) scales and social, clinical variables and health status.

Variable		1-Feeling Understood and Supported by Healthcare Providers	2-Having Sufficient Information to Manage My Health	3-Actively Managing My Health	4-Social Support for Health	5-Appraisal of Health Information	6-Ability to Actively Engage with Healthcare Providers	7-Navigating the Healthcare System	8-Ability to Find Good Health Information	9-Understand Health Information Well Enough to Know What to Do
	Simple Size (*n*)	Mean (SD)	Mean (SD)	Mean (SD)	Mean (SD)	Mean (SD)	Mean (SD)	Mean (SD)	Mean (SD)	Mean (SD)
**Sex**	453									
Female	221	3.20 (0.48)	3.21 (0.45)	2.83 (0.46)	3.04 (0.53)	2.80 (0.49)	3.82 (0.63)	3.58 (0.6)	3.49 (071)	3.67 (0.66)
Male	232	3.21 (0.45)	2.97 (0.47)	2.82 (0.46)	3.11 (0.46)	2.82 (0.46)	3.89 (0.55)	3.61 (0.55)	3.56 (0.60)	3.70 (0.59)
ANOVA F (*p* value)		0.063 (0.802)	0.698 (0.493)	0.263 (0.793)	−1.316 (1.189)	−0.364 (0.716)	−1.042 (0.98)	−0.601 (0.548)	−1.018 (0.309)	−0.528 (0.598)
ES (95% CI) ^a^		0.00 (0.00–0.00)	0.001 (0.000–0.002)	0.00 (0.000–0.000)	0.004 (0.002–0.008)	0.000 (0.000–0.000)	0.002 (0.001–0.005)	0.001 (0.000–0.002)	0.002 (0.001–0.005)	0.001 (0.000–0.001)
**Age**	453									
18–44	77	3.42 (0.47)	3.21 (0.48)	2.92 (0.48)	3.24 (0.45)	3.05 (0.44)	3.99 (0.50)	3.83 (0.48)	3.91 (0.421)	3.92 (0.43)
45–64	198	3.15 (0.45)	2.94 (0.45)	2.77 (0.48)	3.02 (0.48)	2.88 (0.46)	3.84 (0.63)	3.51 (0.61)	3.54 (0.68)	3.72 (0.64)
≥65	178	3.17 (0.46)	2.93 (0.49)	2.85 (0.43)	3.07 (0.50)	2.73 (0.47)	3.81 (0.58)	3.54 (0.57)	3.34 (0.65)	3.56 (0.64)
ANOVA F (*p* value)		10.452 (0.000) **	10.401 (0.000) **	3.475 (0.032) *	5.254 (0.006) *	13.816 (0.000) **	2.761 (0.064)	8.308 (0.000) **	21.539 (0.000) **	9.876 (0.000) **
ES (95% CI) ^a^		0.044 (0.013–0.084)	0.045 (0.011–0.044)	0.015 (0.004–0.015)	0.023 (0.006–0.022)	0.058 (0.015–0.058)	0.012 (0.003–0.012)	0.036 (0.009–0.035)	0.087 (0.02–0.087)	0.042 (0.011–0.042)
**Education level**	451									
<4 years of school	135	3.18 (0.42)	2.92 (0.45)	2.84 (0.45)	3.02 (0.52)	2.69 (0.41)	3.78 (0.58)	3.44 (0.56)	3.19 (0.67)	3.36 (0.66)
4–9 years of school	99	3.11 (0.46)	2.93 (0.46)	2.78 (0.43)	3.04 (0.47)	2.74 (0.44)	3.81 (0.66)	3.52 (0.68)	3.47 (0.66)	3.96 (0.60)
10–12 years of school	108	3.20 (0.49)	2.99 (0.50)	2.76 (0.47)	3.09 (0.51)	2.88 (0.48)	3.91 (0.57)	3.67 (0.55)	3.63 (0.57)	3.81 (0.56)
University	109	3.32 (0.47)	3.09 (0.50)	2.92 (0.49)	3.16 (0.48)	2.94 (0.45)	3.93 (0.57)	3.77 (0.50)	3.89 (0.52)	3.97 (0.46)
ANOVA F (*p* value)		3.776 (0.011) *	3.047 (0.029) *	2.716 (0.044) *	1.738 (0.158)	7.369 (0.000) **	1.805 (0.145)	7.954 (0.000) **	27.610 (0.000) **	24.052 (0.000) **
ES (95% CI) ^a^		0.025 (0.004–0.028)	0.020 (0.003–0.013)	0.018 (0.003–0.012)	0.012 (0.002–0.008)	0.048 (0.008–0.031)	0.012 (0.002–0.008)	0.051 (0.087–0.034)	0.156 (0.03–0.109)	0.139 (0.029–0.097)
**Employment status**	452									
Employed	184	3.23 (0.47)	3.00 (0.49)	2.79 (0.46)	3.14 (0.45	2.84 (0.47)	3.93 (0.57)	3.68 (0.55)	3.68 (0.60)	3.81 (0.59)
Retired	213	3.19 (0.47)	2.96 (0.47)	2.86 (0.46)	3.07 (0.52)	2.75 (0.48)	3.80 (0.60)	3.51 (0.59)	3.36 (0.69)	3.55 (0.66)
Other	45	3.15 (0.44)	3.00 (0.46)	2.89 (0.46)	2.91 (0.52)	2.91 (0.48)	3.80 (0.59)	3.59 (0.60)	3.68 (0.58)	3.80 (0.48)
ANOVA F (*p* value)		0.627 (0.535)	0.453 (0.636)	1.279 (0.279)	4.831 (0.008) *	2.984 (0.052)	2.775 (0.063)	4.436 (0.012) *	13.867 (0.000) **	10.565 (0.000) **
ES (95% CI) ^a^		0.015 (0.001–0.005)	0.004 (0.000–0.002)	0.015 (0.002–0.006)	0.023 (0.003–0.016)	0.017 (0.001–0.007)	0.026 (0.003–0.010)	0.022 (0.002–0.009)	0.060 (0.006–0.025)	0.046 (0.005–0.019)
**Cohabitation**										
Living with others	377	3.20 (0.46)	2.99 (0.47)	2.82 (0.45)	3.11 (0.52)	2.81 (0.47)	3.87 (0.63)	3.61 (0.63)	3.55 (0.61)	3.72 (0.59)
Living alone	72	3.21 (0.49)	2.91 (0.55)	2.86 (0.52)	2.90 (0.64)	2.80 (0.51)	3.77 (0.63)	3.50 (0.61)	3.40 (0.76)	3.55 (0.75)
ANOVA F (*p* value)		0.00 (0.999)	1.503 (0.059)	−0.722 (0.372)	3.342 (0.001) *	0.218 (0.946)	1.324 (0.232)	1.428 (0.408)	1.734 (0.020) *	2.131 (0.08)
ES (95% CI) ^a^		0.00 (0.000–0.000)	0.008 (0.002–0.008)	0.002 (0.000–0.001)	0.041 (0.011–0.041)	0.001 (0.000–0.001)	0.005 (0.001–0.005)	0.005 (0.001–0.005)	0.007 (0.002–0.007)	0.012 (0.003–0.012)
**Type of Diabetes**										
Type 1 Diabetes	104	3.35 (0.45)	3.15 (0.49)	2.92 (0.46)	3.19 (0.48)	2.93 (0.455)	3.97 (0.51)	3.73 (0.53)	3.79 (0.55)	3.84 (0.48)
Type 2 Diabetes	338	3.16 (0.47)	2.93 (0.47)	2.80 (0.46)	3.04 (0.50)	2.77 (0.47)	3.82 (0.61)	3.55 (0.59)	3.45 (0.67)	3.64 (0.65)
Other types Diabetes	11	3.02 (0.47)	2.98 (0.42)	2.60 (0.43)	3.00 (0.379)	2.89 (0.56)	3.74 (0.49)	3.47 (0.63)	3.38 (0.67)	3.70 (0.36)
ANOVA F (*p* value)		7.503 (0.001) *	8.455 (0.000) **	3.779 (0.24) *	3.815 (0.023) *	4.972 (0.007) *	2.956 (0.053)	4.206 (0.016) *	11.236 (0.000) **	4.486 (0.012) *
ES (95% CI) ^a^		0.032 (0.008–0.032)	0.037 (0.009–0.036)	0.017 (0.004–0.016)	0.017 (0.004–0.017)	0.022 (0.006–0.022)	0.013 (0.003–0.013)	0.012 (0.005–0.018)	0.048 (0.012–0.047)	0.020 (0.005–0.019)
**Hemoglobin glycated (A1c)**										
<6.5%	57	3.31 (0.47)	3.06 (0.51)	2.95 (0.52)	3.24 (0.41)	2.87 (0.43)	2.95 (0.53)	3.68 (0.59)	3.64 (0.60)	3.70 (0.67)
6.5–7.4%	114	3.21 (0.51)	3.02 (0.47)	2.92 (0.46)	3.06 (0.53)	2.89 (0.51)	2.92 (0.47)	3.61 (0.57)	3.58 (0.68)	3.79 (0.56)
7.5–8.4%	114	3.24 (0.44)	2.99 (0.47)	2.81 (0.41)	3.09 (0.46)	2.79 (0.50)	3.83 (0.41)	3.54 (0.55)	3.51 (0.61)	3.64 (0.63)
≥8.5%	156	3.12 (0.45)	2.92 (0.48)	2.72 (0.46)	3.03 (0.49)	2.75 (0.45)	2.72 (0.46)	3.58 (0.60)	3.47 (0.68)	3.67 (0.62)
ANOVA: F (*p* value)		2.823 (0.038) *	1.480 (0.219)	5.849 (0.001) *	2.660 (0.048) *	2.366 (0.070)	0.252 (0.860)	0.798 (0.496)	1.130 (0.336)	1.204 (0.308)
ES (95% CI) ^a^		0.010 (0.02–0.010)	0.005 (0.001–0.005)	0.026 (0.007–0.026)	0.016 (0.004–0.016)	0.008 (0.002–0.008)	0.001 (0.000–0.001)	0.003 (0.000–0.003)	0.007 (0.002–0.007)	0.007 (0.002–0.006)
**Years living with diabetes**	453									
<10	108	3.24 (0.49)	2.93 (0.48)	2.86 (0.53)	3.05 (0.56)	2.86 (0.52)	3.89 (0.57)	3.68 (0.56)	3.64 (0.65)	3.78 (0.65)
10–20	168	3.15 (0.43)	2.95 (0.47)	2.74 (0.44)	3.03 (0.45)	2.75 (0.45)	3.84 (0.57)	3.55 (0.55)	3.74 (0.64)	3.67 (0.57)
>20	177	3.25 (0.49)	3.05 (0.48)	2.90 (0.43)	3.15 (0.50)	2.84 (0.47)	3.85 (0.63)	3.58 (0.63)	3.51 (0.68)	3.65 (0.65)
Mean (SD)		3.25 (0.49)	3.05 (0.48)	2.90 (0.43)	3.15 (0.50)	2.84 (0.47)	3.85 (0.63)	3.58 (0.63)	3.51 (0.68)	3.65 (0.65)
ANOVA: F (*p* value)		2.523 (0.081)	2.633 (0.073)	5.478 (0.004) *	2.624 (0.074)	2.296 (0.102)	2.296 (0.754)	1.673 (0.189)	2.191 (0.113)	1.420 (0.243)
ES (95% CI) ^a^		0.011 (0.003–0.011)	0.016 (0.003–0.011)	0.024 (0.006–0.023)	0.012 (0.00–0.012)	0.010 (0.003–0.01)	0.005 (0.000–0.003)	0.009 (0.002–0.006)	0.010 (0.002–0.010)	0.006 (0.002–0.006)
**Self-reported health status**										
Good	117	3.28 (0.47)	3..10 (0.50)	2.99 (0.47)	3.17 (0.50)	2.95 (0.48)	3.99 (0.54)	3.83 (0.54)	3.81 (0.62)	3.97 (0.51)
Fair	243	3.17 (0.45)	2.97 (0.46)	2.79 (0.44)	3.06 (0.48)	2.77 (0.45)	3.86 (0.59)	3.56 (0.56)	3.50 (0.63)	3.67 (0.60)
Poor	91	3.16 (0.49)	2.85 (0.47)	2.71 (0.44)	2.97 (0.51)	2.72 (0.46)	3.66 (0.61)	3.36 (0.58)	3.22 (0.67)	3.67 (0.40)
ANOVA: F (*p* value)		2.486 (0.084)	7.353 (0.001) *	11.374 (0.000) **	4.420 (0.013) *	7.864 (0.000) **	8.333 (0.000) **	18.090 (0.000) **	22.932 (0.000) **	23.740 (0.000) **
ES (95% CI) ^a^		0.011 (0.003–0.010)	0.032 (0.008–0.031)	0.049 (0.012–0.049)	0.019 (0.005–0.019)	0.034 (0.009–0.034)	0.036 (0.009–0.036)	0.075 (0.012–0.074)	0.093 (0.025–0.092)	0.107 (0.029–0.106)

* *p* < 0.05; ** *p* < 0.001. ^a^ Statistically significant difference bolded.

## Data Availability

Not applicable.
